# Lifelong Personalization *via* Gaussian Process Modeling for Long-Term HRI

**DOI:** 10.3389/frobt.2021.683066

**Published:** 2021-06-07

**Authors:** Samuel Spaulding, Jocelyn Shen, Hae Won Park, Cynthia Breazeal

**Affiliations:** Massachusetts Institute of Technology, Cambridge, MA, United States

**Keywords:** transfer learning, lifelong learning, continual learning, user modeling, human-robot interaction, educational games, Gaussian processes, adaptive personalization

## Abstract

Across a wide variety of domains, artificial agents that can adapt and personalize to users have potential to improve and transform how social services are provided. Because of the need for personalized interaction data to drive this process, long-term (or longitudinal) interactions between users and agents, which unfold over a series of distinct interaction sessions, have attracted substantial research interest. In recognition of the expanded scope and structure of a long-term interaction, researchers are also adjusting the personalization models and algorithms used, orienting toward “continual learning” methods, which do not assume a stationary modeling target and explicitly account for the temporal context of training data. In parallel, researchers have also studied the effect of “multitask personalization,” an approach in which an agent interacts with users over multiple different tasks contexts throughout the course of a long-term interaction and learns personalized models of a user that are *transferrable* across these tasks. In this paper, we unite these two paradigms under the framework of “Lifelong Personalization,” analyzing the effect of multitask personalization applied to dynamic, non-stationary targets. We extend the multi-task personalization approach to the more complex and realistic scenario of modeling dynamic learners over time, focusing in particular on interactive scenarios in which the modeling agent plays an active role in teaching the student whose knowledge the agent is simultaneously attempting to model. Inspired by the way in which agents use active learning to select new training data based on domain context, we augment a Gaussian Process-based multitask personalization model with a mechanism to actively and continually manage its own training data, allowing a modeling agent to remove or reduce the weight of observed data from its training set, based on interactive context cues. We evaluate this method in a series of simulation experiments comparing different approaches to continual and multitask learning on simulated student data. We expect this method to substantially improve learning in Gaussian Process models in dynamic domains, establishing Gaussian Processes as another flexible modeling tool for Long-term Human-Robot Interaction (HRI) Studies.

## 1 Introduction

Our goal is to develop adaptive robotic agents that are deeply personalized and designed for long-term interaction. Such an agent would be an invaluable asset that could foster learning in ways similar to those of the best human teachers, yet still provide the advantages of digital technology such as data fluency, always-on availability, and scale of distribution. Educational researchers have long recognized that a *personalized* approach to pedagogy is one of the best ways of promoting learning (Pane et al., 2015), yet in a world with increasing demand for education, the availability of qualified teachers has not kept up with the demand from students. Technology has an important role to play in realizing the vision of personalized education for all. In recognition that learning is not only a cognitive process, but also an emotional and a social process, we seek to design *social robots learning companions* that are capable of understanding students, adapting to them, and introducing them to educational material that is best suited for each student, presented in a way that takes into account their individual learning differences.

A 2018 review (Belpaeme et al., 2018) on the use of social robots as educational tools concluded “(social robots) have been shown to be effective at increasing cognitive and affective outcomes and have achieved outcomes similar to those of human tutoring *on restricted tasks*” (emphasis ours). What are these restricted task scenarios? “short, well-defined lessons delivered with limited adaptation to individual learners or flexibility in curriculum.” Results from studies of single-session tutoring interactions with limited personalization paint an overall picture of benefits that are stable, positive, and modest. In order to improve the impact of social robot tutoring technology, researchers are looking toward educational interactions where personalization plays a larger role, and to long-term interactions to develop deeply personalized models.

Despite general recognition that long-term interactions enable a more impactful approach for the field, developing agents capable of sustaining long-term interactions is no simple feat. Some of the challenges researchers face in sustaining long-term interactions include lower student engagement (due to repetitive interactions and declining novelty), personalized models that represent only limited aspects of student mastery (narrowly focused models are more straightforward to implement and require less data to train), and early stopping (due to cold-start model learning, leading to poor model performance in early sessions).

### 1.1 Multitask Personalization - Learning Personalized Models Across Different Task Contexts

To address some of these challenges, we advocate an approach to long-term interaction design called “multi-task personalization” in which students interact with a social robot across different task contexts throughout a long-term interaction. Within each task, the robotic interaction partner learns a task-specific personalized model of the student that is *transferrable* across tasks throughout the long-term interaction, i.e., data collected from earlier interactions with a student on a prior task can be used to improve personalized model learning in a new task.

A multitask personalization approach has potential to address many of the practical challenges associated with sustaining long-term interactions. Student engagement is likely to remain higher over time when engaged in different, varied tasks with a learning partner, compared to repeating the same task multiple times. Personalized student models can also draw on data from a wider variety of task contexts in order to learn a more multifaceted picture of a student’s mastery. And transferring data from interactions on prior tasks can help speed up model learning on a new task, reducing the risk of early stopping from cold-start learning.

In prior work (Spaulding et al., 2021) we laid out the theoretical benefits of a multi-task personalization paradigm and evaluated the combined-task proficiency and data efficiency of the approach in models trained to estimate simulated student mastery in two different game tasks. These games, called RhymeRacer and WordBuilder, were developed in partnership with experts in children’s media and early literacy learning, and were designed to help young students practice different literacy skills, namely rhyming and spelling.

We developed a flexible Gaussian Process-based approach to modeling student knowledge in each game task, with an instance-weighting protocol based on task similarity that allowed for data transfer across tasks. We showed that multi-task personalization improved the sample-efficiency of model training, and was particularly useful for avoiding the problem of “cold-start” modeling. This research was conducted with the assumption that student knowledge was static i.e., that students’ level of knowledge was fixed throughout the interaction sequence. In order to further validate the potential of multitask personalization for real-world scenarios—and recognizing that in real human-robot educational interactions, a student is not a fixed target but a dynamic one—we look to augment our original approach to multitask personalization with methods that better support personalized modeling of dynamic/non-stationary targets.

### 1.2 Continual Learning-Learning Personalized Models of Non-Stationary Targets Over Time

“Continual Learning” (CL) – a “learning paradigm where the data distribution and learning objective change through time, or where all the data … are never available at once” (Lesort et al., 2020) is precisely the family of methods to complement our original multitask personalization approach. Continual Learning primarily deals with the issue of distributional *shift* over time, recognizing that, in the real world, temporal data are not independent and identically distributed (“i.i.d”), but rather drawn from a distribution that may change over time, but without a clear signal of such a shift (Lesort et al., 2020). Continual Learning techniques attempt to improve model performance as this shift occurs, often with an implicit assumption that such shifts will be relatively smooth.

Multitask learning, on the other hand, focuses more on learning distinct tasks with clear boundaries. In a typical multitask learning scenario, a learner knows from which tasks its training data originated, assumes that each such task is stationary and that its data is *i.i.d*, and, frequently, the training data arrive in a batch, rather than over time. These broad distinctions can largely be characterized by a focus on task “shift” *vs.* task “switch” However, this boundary is not always strict, and researchers often work to address both issues simultaneously [e.g., (Ruvolo and Eaton, 2013)].

As human-robot interaction (HRI) researchers have begun to adapt research methods towards long-term interactions, continual learning methods have become more popular in the algorithms and models underlying these interactions. Churamani et al. have detailed many advantages of adopting a continual learning approach in developing affect-aware interactive robots (Churamani et al., 2020). The authors highlight a number of important shifts in viewpoint when adopting this approach-recognizing that human affective response is idiosyncratic (i.e., personalized), dynamic (i.e., changes over time), and contextual (i.e., changes with task or environment). We argue that these same qualities apply more broadly, to many aspects of human interactive behavior, though in this paper we primarily focus on student learning in educational interactions. Indeed, many of the most salient markers of learning behavior *are* affective behaviors, therefore it is only a short conceptual leap to hypothesize that the benefits of Continual Learning applied to affect recognition and response may prove similarly beneficial when applied to recognizing and responding to student learning behaviors.

Though Churamani et al. did not explicitly refer to *long-term* interactions (LTI) with users, the theoretical frameworks of continual learning and multitask personalization are natural fits for the practical goal of sustaining long-term interactions. Our goal in this paper is to demonstrate the strengths of this combined approach by emphasizing their benefits in the application domain of an agent attempting to model student knowledge in the form of a StudentModel. Students’ knowledge is idiosyncratic (each student has their own private mastery model), dynamic (this model can change over the course of an interaction), and contextual (student knowledge can manifest differently in different task contexts). A modeling approach that acknowledges and accounts for these qualities may be the key to successful, personalized long-term interactions.

### 1.3 Lifelong Personalization-Personalized Modeling Across Tasks and Over Time

Therefore, we propose to move beyond the traditional algorithmic view of modeling student knowledge as supervised learning of a fixed target, or “estimation” of mastery on a single task. Instead, we adopt a broader view of student modeling that incorporates ideas from both continual and multitask learning into an approach to long-term student modeling as a process of personalization *over time* and *across tasks*, which we refer to as “lifelong personalization.”

To motivate our use of this term and connect the dots between various methods referred to in other literature, we outline here the relational structure of several key concepts used throughout the remainder of the paper.

**FIGURE 1:**
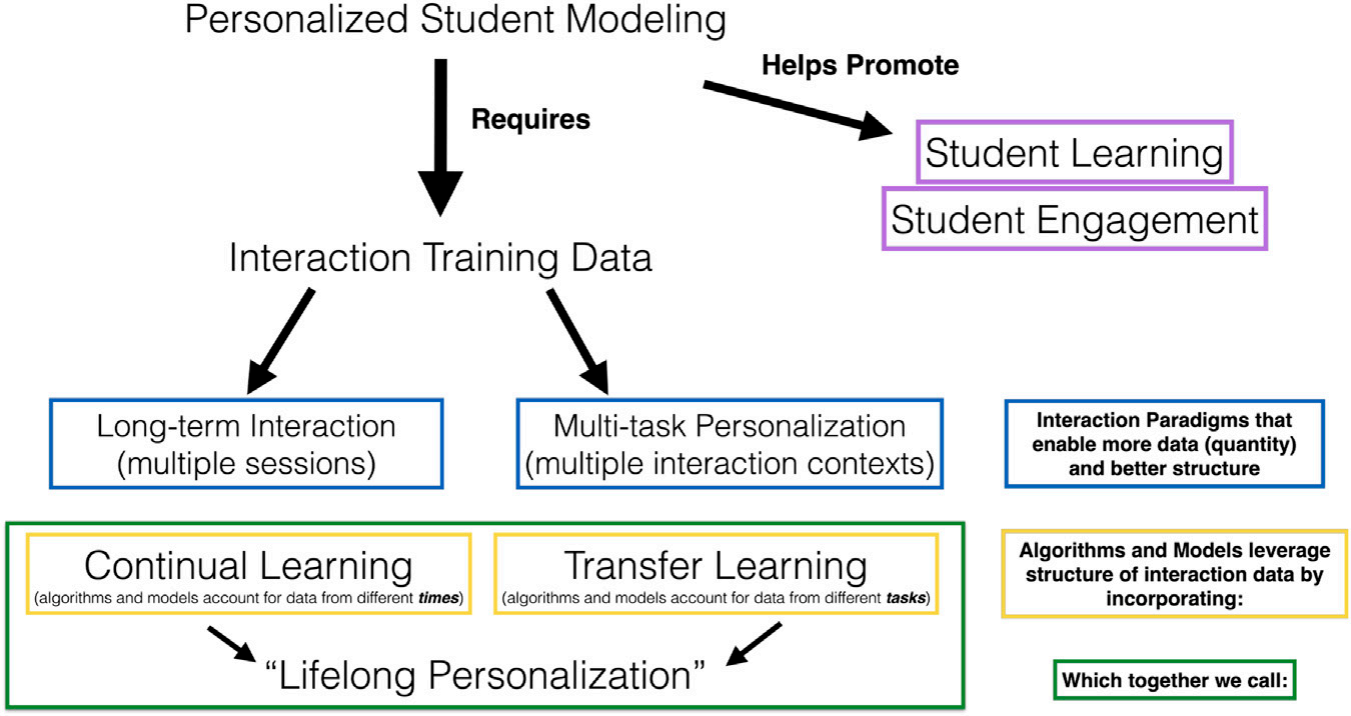
Conceptual structure of terms and goals.

Personalized student modeling has been shown to help promote student learning and engagement (Yudelson et al., 2013; Lindsey et al., 2014; Park et al., 2019; Ramachandran et al., 2019). In order to advance the degree and sophistication of personalized modeling, we require personalized interaction data from a student. To elicit useful quantities and kinds of personalized student data, researchers have been looking towards long-term interaction designs (Leite et al., 2013), which occur over several sessions at different *times*. After observing shortcomings of single-task longitudinal interactions, we introduced the idea of “multi-task personalized” interactions, which occur in different *task contexts* (Spaulding et al., 2021). Each of these paradigm shifts in interaction design are mirrored by an associated paradigm shift in algorithm and model design: continual learning, which accounts for the temporal sequence in which data is received and assumes a dynamic or non-stationary modeling target, and transfer learning which accounts for the task in which training data originated and uses data from one “source” task to more quickly learn a model in a different “target” task. When we combine these two algorithmic paradigms, yielding flexible personalized models that can model individuals over time and across tasks, we call this lifelong personalization, based on Parisi et al.’s definition of lifelong learning systems as “an adaptive algorithm capable of learning from a continuous stream of information, with such information becoming progressively available over time and where the number of tasks to be learned (e.g., membership classes in a classification task) are not predefined” (Parisi et al., 2019). This concept structure is represented graphically in Figure 1.

Of course, these are not universal definitions of these terms, and many researchers may interpret or use these terms in slightly different ways. In this paper, we apply these definitions in the context of *personalized interactive modeling*. Some works look at separate individuals as separate tasks (Jaques et al., 2017), some works consider non-stationary task learning as the primary hallmark of lifelong learning (Xie et al., 2020). For the purposes of this paper, however, we restrict our discussion to the application of these paradigms in the context of learning models of an individual over time and across tasks for *adaptive personalization*.

### 1.4 Research Contributions

Our main experimental contributions in this paper include an expanded treatment of prior work on multitask personalization, evaluating an approach to transferrable player models based on Gaussian Processes in a simplified modeling scenario that assumes student knowledge does not change throughout an interaction. We then extend this approach to a more complex, nonstationary simulation scenario that incorporates the in-game *actions* of the tutoring agent, and the effect of these actions on the underlying knowledge of the student modeling target (i.e., student learning in response to tutoring actions).

We show that ignoring the effect of in-game tutoring actions on student knowledge (by assuming a stationary modeling target) reduces the accuracy of the agent’s learned student model, but that by augmenting the agent with a mechanism for *continual active training data management* (see below), the learned student models can account for context-dependent shifts in the students underlying knowledge and learn more accurate final models of student knowledge. When we discuss “adaptive personalized tutoring agents,” we fundamentally mean developing *agents that select tutoring actions based on a personalized student model*. By extending our simulated evaluation to scenarios that account for the impact of tutoring agent *actions*, not only can we study the effect of multitask and continual learning methods on tutoring agents’ learning of personalized student models, but we can also study how these methods ultimately impact *student* learning, by tracking the degree of shift in underlying student knowledge.

In addition to experimental work exploring core questions of viability, efficiency, and proficiency benefits provided by multi-task personalization in nonstationary environments (i.e., “lifelong personalization”), another goal of this article is to further stimulate discussion on the algorithmic impact and interaction design considerations of multitask personalization, bringing together perspectives on Continual Learning with an applied focus on practical goals of HRI experimenters, through the lens of educational interactions. In particular, we advocate for more widespread simulation analysis of students in personalized algorithmic or model-driven long-term HRI work. Given the difficulty and complexity of deployed long-term HRI studies, and the potential for simulation research to assist in tuning hyperparameters or validating algorithmic approaches, we advocate for establishing simulation results as a “best practice” benchmark before undertaking in-person studies.

### 1.5 Summary of Approach

This paper directly extends prior research on multitask personalization. In Spaulding et al. (2021) we conducted a study of multitask personalization paradigm by evaluating the effects of taking personalized training data from the StudentModel of one game and transferring it to train the StudentModel of the other using an instance-weighting scheme based on task-similarity. Based on simulation data from a simplified model of a student, we showed that this form of multitask personalization was viable (i.e., supported transfer between source and target tasks without negatively impacting target task performance) and improved data efficiency (i.e., showed clear evidence of avoiding cold-start learning in the target task), especially early in the target task.

**FIGURE 2:**
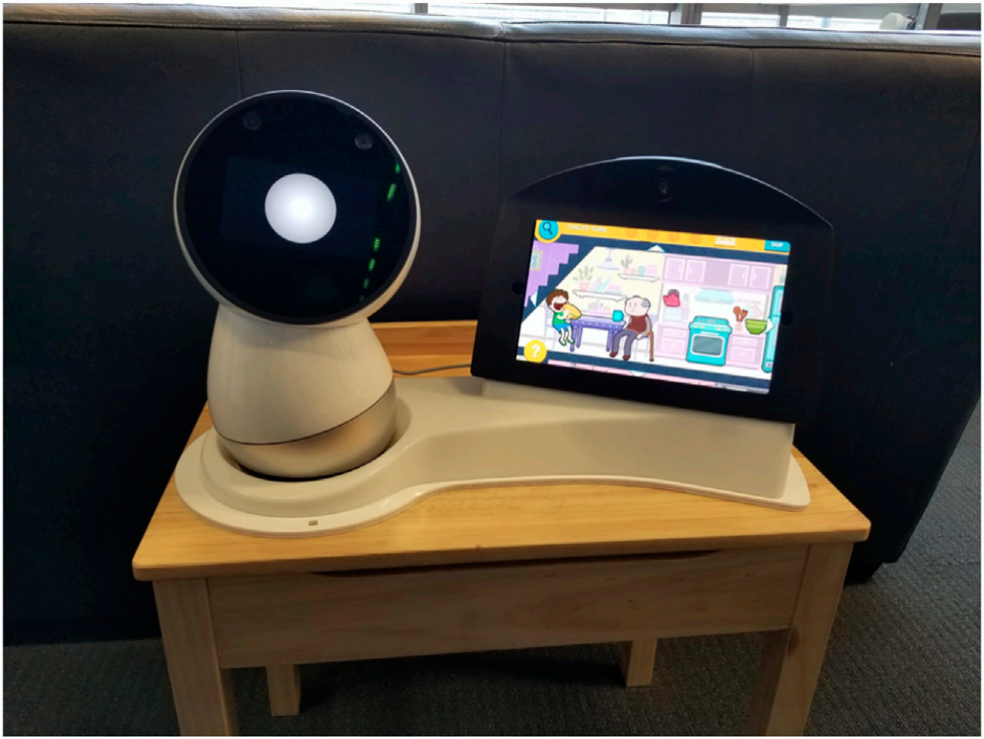
An integrated social robot platform that supports different game “tasks.”

The simulated student data was derived from automated playthroughs of two interactive game tasks called RhymeRacer and WordBuilder, part of an integrated physical system that supports educational co-play between live students and a social robot (see Figure 2). Both games were designed in collaboration with experts in early literacy and children’s media to help young students practice literacy and language skills. Within each game, a student and a robotic tutoring agent play together, answering puzzles designed to promote early literacy and language skills, such as rhyming and spelling. Throughout each game, the robotic agent attempts to learn a StudentModel of the student player’s mastery of the game, based on their in-game actions and responses. This StudentModel is then used within each game to adapt content and robot tutoring actions to improve student learning in a personalized way.

In Spaulding et al. (2021), we were primarily concerned with understanding and evaluating basic performance of multitask personalization. In our simulated playthroughs, we made a number of simplifying assumptions, ignoring actions of the robot tutor player and assuming that the student’s underlying mastery of words (i.e., state of knowledge), though observed noisily, was fixed. Those assumptions ensured that the distribution of each individual student’s responses was IID. In this paper, we take another step toward more realistic interactions and present results from simulation experiments that relax those assumptions. We present results from a new round of simulation experiments, in which the robotic agent now takes an additional active role in the interaction, selecting words to “demonstrate” to the student as a lesson, which stochastically alters the student’s underlying mastery (which is later noisily observed by the tutoring agent). As demonstrations shift the student’s mastery over time within each task, the proficiency of each tasks’ StudentModel declines, presenting an opportunity to adopt a continual learning approach alongside multitask personalization.

The computational tool underlying each game’s StudentModel is a Gaussian Process (GP). Past studies have shown that Gaussian Processes can serve as a flexible paradigm for modeling user’s knowledge state in interactive learning scenarios (Griffiths et al., 2009; Spaulding et al., 2018). Gaussian Processes tend to be data-efficient relative to other supervised learning methods (e.g., deep learning), and domain knowledge such as connections between curricular components can be encoded *via* the covariance kernel that drives the GP inference. Over several prior projects, we have developed a unified “word-space” representation for Gaussian Processes that has successfully been used to model student mastery over a wide variety of different language and literacy skills (Spaulding et al., 2018; Spaulding and Breazeal, 2019; Spaulding et al., 2021). Our approach to multitask personalization involves transferring the student data used to train each task’s StudentModel from its originating (source) task to another (target) task, re-weighting each observed point based on an instance-specific measure of task similarity derived from each task’s Gaussian Process covariance kernel (see Section 3.4 for more detail).

One drawback of Gaussian Processes, however, is that they do not naturally have a representation of data received over time (in contrast to Dynamic Bayesian Network approaches like Knowledge Tracing). Because of this, when modeling a dynamic target, such as a student learner or other non-stationary domain, Gaussian Processes are slower to adjust to shifts in the data generation process, compared to learners which weight recent data more heavily.

To improve the ability of the GP-based StudentModels to handle lifelong personalization, we introduce a *continual active training data management* (**CATDaM**) mechanism that allows the tutoring agent to proactively manage its training data through a novel two-way active learning protocol, enabling the model to both select new data points to add to its training set and automatically prune its existing training set to remove “stale” data points that may no longer be a good representation of the dynamic student. The normal (mean and variance) form of the Gaussian Process posterior distribution at each domain point has a natural interpretation as a model estimate and uncertainty surrounding that estimate, another way in which Gaussian Processes are well-suited for agent-based machine learning settings. The tutoring agent steers student learning by proactively choosing words to demonstrate (i.e., teach a lesson about) to the student based on the latest StudentModel, and actively improves StudentModel learning by proactively choosing new training data to observe or by deleting old training data that may no longer reflect the student’s shifted mastery.

We find that the sample-efficiency benefits of multitask personalization are largely preserved, but that both single-task and multitask GP models that do not use **CATDaM** show a substantial drop in performance in the non-stationary setting. However, both single-task and multitask GP models that do use **CATDaM** regain and/or exceed this performance drop in the more complex non-stationary environment. Moreover, because we are now modeling the impact of tutor actions on dynamic students, we can evaluate the impact of algorithm and model changes on estimated *student* learning, in addition to model learning. We find that in interaction with agents whose StudentModels use **CATDaM**, students learned (underlying mastery went from negative to positive as a result of an agent demonstration) approximately 50% more words. Together these results suggest substantial benefits to adopting a “lifelong personalization” approach — *via* instance-weighted data transfer for multitask personalization and **CATDaM** for continual learning — to long-term human-robot interaction.

## 2 Related Work

### 2.1 Perspectives on Lifelong Personalization

Long-term or *Longitudinal Interaction* (LTI) is a term used to refer to interactions between a user and an artificial agent that unfold over multiple distinct encounters (Irfan et al., 2019). In other words, “long-term interaction” describes a practical paradigm for designing and evaluating interactions between users and agents. In the context of educational interactions, long-term interactions have followed a pattern of users engaging in a single repeated interaction structure (i.e., playing a single game or answering questions) with updated content reflecting the output of increasingly personalized models trained on data from the previous interaction sessions (Leite et al., 2013). While this type of repeated single-task interaction has formed the bulk of long-term interaction research to date, there is a recognition that we may be near the useful limit of current single-task paradigms, and that future breakthroughs in sustaining long-term interactions will come from research developing agents that can personalize to a user’s changing behaviors and preferences over time and across task contexts.

Johnson and Lester, in an article reflecting on 20 years of research to predict future trends for pedagogical artificial agents wrote: “Conventional domain-specific learner models may be useful for pedagogical agents in the short term, but they will be of limited value over time as learners move between learning experiences (Johnson and Lester, 2018).”

Melanie Mitchell, weighing in on the utility of modern AI systems, wrote:

In fact, the theoretical basis for much of machine learning requires that training and test examples are “independently and identically distributed” (IID). In contrast, human learning—and teaching—is active, sensitive to context, driven by top-down expectations, and transferable among highly diverse tasks, whose instances may be far from IID (Mitchell, 2020).

Finally, in a lecture addressing future challenges for the field of Learning Analytics, Ryan Baker identified “*transferability*” as the first of a series of challenge problems for the field to tackle over the next 20 years, writing.

A modern learning system learns a great deal about a student—their knowledge at minimum, and increasingly their motivation, engagement, and self-regulated learning strategies. But then the next learning system starts from scratch … It’s like there’s a wall between our learning systems … If you seek better learning for students, tear down this wall (Baker, 2019)

Fundamentally, personalized student data remains a major practical challenge towards achieving successful interactive educational systems. Single-session educational interactions in HRI [some, e.g., reviewed in (Belpaeme et al., 2018)] generally do not provide enough data to learn interesting and distinct personalized models capable of sustaining extensive learning gains or engaged interaction in the long-term. Thus far, successful examples of long-term adaptive personalization tend to repeat a carefully designed interaction centered on a single task over several sessions to augment the dataset (Park et al., 2019; Ramachandran et al., 2019).

### 2.2 Related Work on Transfer Learning and Nonstationary Modeling in Gaussian Processes

In general, rather than compiling lists of relevant citations, we prefer to introduce and cite prior work at relevant sections throughout this paper. However, owing to the more abstract nature of the following articles and less *direct* applicability to the empirical content of the rest of the paper, we wish to briefly highlight some particularly helpful articles that inspired our thinking in the area of transfer learning and nonstationary modeling, as applied to Gaussian Processes.

Soh et al.’s formulation of transferrable trust models using Gaussian Processes uses a similar kernelized “task” representation to our design of task-specific StudentModels (Soh et al., 2020). Snoeke and Adams outlined an “input-warping” method to address nonstationarity in Gaussian Processes that provided a clear exposition of theoretical capabilities of GPs to handle nonstationary functions (Snoek et al., 2014). Cao et al. introduced us to the idea of transfer-coefficient based instance-weighting for Gaussian Processes (Cao et al., 2010), and our evaluation measures of transfer viability, efficiency, and proficiency are based on discussion in Rosenstein et al. (2005).

## 3 Personalized Literacy Game System

To investigate the algorithmic effects of multitask personalization and lifelong learning in students, we have developed an integrated, deployable social robot system capable of sustaining language/literacy practice between young students and a robot through game-based interactions. Thus far, we have used this system to investigate multitask personalization *via* player model transfer between two games, called RhymeRacer and WordBuilder, which are designed to help young students practice rhyming and spelling skills, respectively, through interactive co-play with an adaptive, personalized robot tutoring agent. Both games were developed for Android tablets using the Unity game engine, and receive robot action commands and relay player input through ROS (Quigley et al., 2009) to a backend system controller. The games were developed for children learning to read, approximately ages 5–7, and throughout the design and development process we consulted experts in children’s media design and early childhood literacy to ensure that both the content and game designs would be age-appropriate and aligned with the overall educational goals of the project.

As the child and robot play each game together, the robot tutoring agent learns a Gaussian Process model, which we refer to as the StudentModel, that estimates the child’s “mastery” of the game. Both games share a Curriculum of words, which serves as both a list of words a student can encounter in the game as well as a unified domain space for the underlying StudentModels of each game. In other words, the StudentModel is an estimate of how likely the student is to successfully apply the primary literacy skill (rhyming, spelling) to each word in the Curriculum, based on observations of their prior gameplay. Each game has undergone playtesting validation and the Curriculum was curated by experts in early childhood learning to ensure a representative set of 74 words that are generally phonetically, orthographically, and semantically (e.g., animals, foods, household items) age-appropriate, and form distinct rhyme groups.

### 3.1 RhymeRacer: Game Design and Educational Principles

**FIGURE 3:**
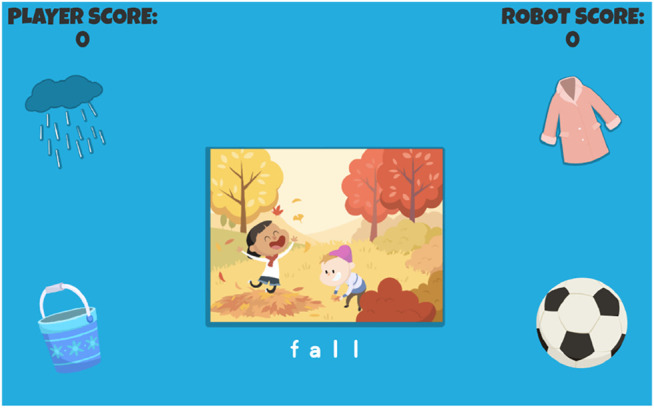
A round of RhymeRacer. FALL is the Target word, Prompt words are RAIN, COAT, PAIL, and BALL.

The gameplay and design of RhymeRacer centers around the family of literacy skills known as “phonological awareness and phonemic articulation.” It is a two-player game that proceeds in a series of rounds, each of which offers a chance for either the robot tutor or the student to select a word that rhymes with a central “prompt word”. In this paper, the gameplay of RhymeRacer is unchanged from its presentation in Spaulding et al. (2021), which we here quote:

“RhymeRacer is … a fast-paced, competitive, 2-player game that proceeds through a series of discrete game rounds. At the start of each round, the tablet shows a picture of the “Target” word in the center of the screen (see Figure 3), surrounded by four “Prompt” word graphics, smaller pictures of other words from the Curriculum, exactly one of which rhymes with the Target word. The tablet also gives a recorded audio prompt, saying “What rhymes with(Target Word)?” as the images are displayed. The first player to correctly tap on the rhyming Prompt word graphic is awarded points, after which the graphics clear and the next round begins.

The robot player is presented to the human player as a co-playing peer, and its outward behavior affirms this framing: the robot player selects Prompt word graphics just as the human player does, gives a mixture of correct and incorrect responses, and responds with appropriate socio-emotional behaviors to in-game events (e.g., acts excited when scoring points, disappointed when incorrect, encouraging when human player scores points).”

### 3.2 WordBuilder: Game Design and Educational Principles

WordBuilder is the second game we developed to study multitask personalization in long-term interactions. It was specifically designed to complement RhymeRacer and went through a similar design process, including playtesting, consultation with educational experts, and content and asset revision by experienced children’s media designers. Most of the visual assets are shared across both games, including the graphics of the Curriculum words, both to help reinforce students’ understanding, and also, practically, to help ensure that the correlation between student performance in the two games is based on students’ mastery of the underlying skills, not on factors related to the game interface design. The gameplay of WordBuilder is also unchanged from its presentation in Spaulding et al. (2021), which we here quote:

“WordBuilder is a brand-new game developed to complement RhymeRacer. The two games use a similar design process and share some game assets to maintain a consistent visual style, most notably the Target word graphics that depict the words from the Curriculum … WordBuilder serves as a counterpart to RhymeRacer in two main ways: First, WordBuilder is designed to help students practice spelling (an alphabetic skill), rather than rhyming (a phonetic skill), to broaden the curricular coverage of the unified system. Second, WordBuilder features collaborative, rather than competitive, gameplay; the robot and child work together to solve a spelling puzzle posed by the tablet, as opposed to the “first-to-answer-wins” style of RhymeRacer.

**FIGURE 4:**
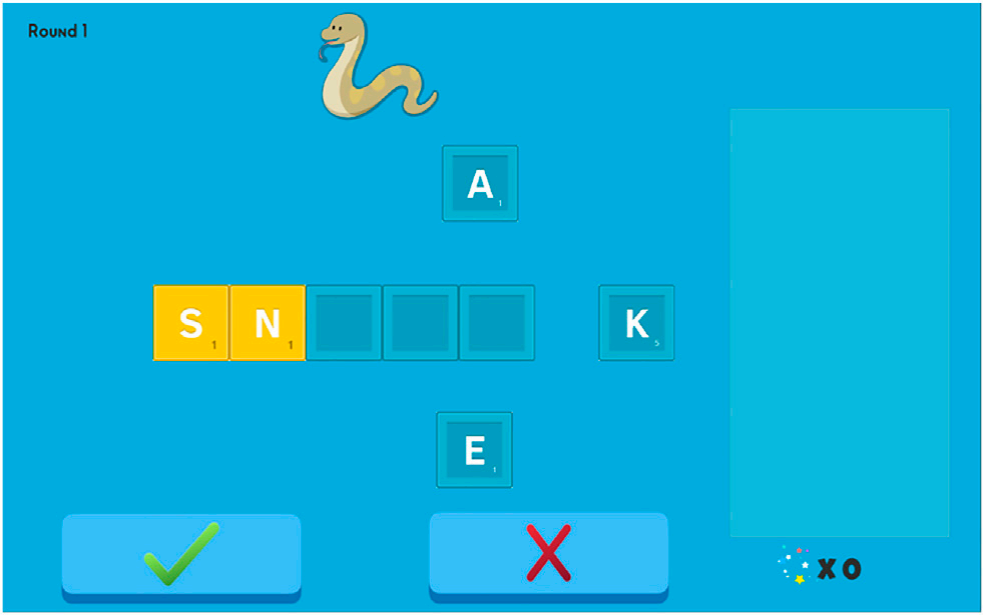
Screencap of a single “round” of WordBuilder. “SNAKE” is the Target word, and the letters “S” and “N” have been provided as a partial solution.

Much like RhymeRacer, gameplay proceeds through a discrete series of rounds, each associated with a round “Target” word whose graphic is displayed at the top of the screen. The letters which make up the Target word are randomly placed into letter blocks surrounding a set of (initially empty) letter slots in the center. For example, if the round Target word is SNAKE, the tablet shows an image of a snake and the letters S-N-A-K-E in letter blocks in a random order and location, surrounding five empty letter slots (see Figure 4). Within each round, the student and the robot can each freely place letter blocks into the center squares to spell words; the round ends when the submit button is pressed, and the human-robot team scores points if the team placed all the letters of the Target word into the correct letter slots. The completed word is then displayed on the right side of the screen, and the next round starts.”

These games were designed together to study multitask personalization in long-term interaction. They share a common software and system architecture and, notably, the word-space Gaussian Process modeling paradigm described in Section 3.4. Within this common framework, however, individual model implementations, like the literacy skills each game is designed to promote, differ considerably.

### 3.3 Strategy and Content Models: Adaptive Gameplay and Content Personalization *Via* Cognitive Modeling

In this paper, we put new emphasis on the robot tutor’s *demonstration actions*. At various points in both games, either the student or the tutor will have an opportunity to respond to a word presented from the Curriculum. The ContentModel determines which specific words, drawn from the Curriculum, are presented, while the StrategyModel determines whether the student is prompted to respond (giving a “sample” of training data for the StudentModel) or whether the robot responds (providing a “demonstration” that can potentially improve student learning). This paradigm of interwoven “samples” and “demonstrations” that mix assessment and learning is an example of the “stealth assessment” design pattern, commonly used to achieve educational goals in interactive games without breaking immersion and experience flow (Shute and Ventura, 2013).

In live gameplay, the robot’s StrategyModel selects probabilistically from two “strategy actions”—OBSERVE and DEMONSTRATE—at each action decision point. When the StrategyModel selects the OBSERVE action, it gives the child an opportunity to respond, prompting a response if none is immediately forthcoming. When the StrategyModel selects the DEMONSTRATE action, the robot proactively gives its own response: a correct answer and an explanation of its reasoning.

The ContentModel determines what specific words from the Curriculum are presented to the players, and in what order. In this work, the ContentModel selects words *via* an Active Learning protocol, which selects the word that best aligns with the goals of the tutor’s selected strategy, given the current estimated StudentModel of the student. For instance, if the current tutor strategy is OBSERVE, the ContentModel selects the word with the maximum uncertainty under the most recent posterior StudentModel i.e., the word where the agent is least confident about its estimate. If the current tutor strategy is DEMONSTRATE, the ContentModel selects the word with lowest variance of all words with negative posterior mean i.e., the word that the agent is *most confident* that the student has *not mastered*.

In order to effectively teach a student, the agent must know what words the student has already mastered and which it has not. Therefore the agent faces the twin challenge of simultaneously estimating a student’s individual knowledge state while using its latest estimate to teach new content, though the act of teaching itself may change the student’s underlying knowledge. The StrategyModel balances these two objectives, while the ContentModel employs active learning to improve both objectives, speeding up both model learning and student learning. As the number of demonstrations increases over time, shifting the student’s knowledge and, therefore, the distribution from which their observed “samples” are drawn, the tutoring agent employs a form of “negative” active learning to *remove* past samples from the StudentModel training set. We expand on the implementation of this ‘continuous active training data management (**CATDaM**) in Section 3.7.

### 3.4 Gaussian Processes: A Flexible Representation for Cognitive Modeling

The fundamental modeling approach we use to represent student’s cognitive task mastery is Gaussian Process regression. A Gaussian Process (GP) is a distribution over functions, defined over some input domain, where the joint distribution of the functions at any finite set of domain points is jointly Gaussian, i.e., at any particular domain point (x∈X), the GP posterior has a Gaussian form (mean and variance), $\left\{\;\mu_{x},\sigma_{x}\right\}$.

A Gaussian Process is defined by a mean function and a covariance function. In discussing GPs, we say that functions, defined over a domain *X*, are distributed according to a Gaussian Process with mean function *μ*, and covariance function *k* (Eq. 1). Functions are sampled (or “realized”) from the GP posterior by combining samples from the GP posterior at a set of domain “test” points (the GP posterior at each point has a normal form). The mean and variance of the GP posterior at each test point is driven by two factors: First, a set of observed training data, $D=\left\{\left\{x_{0},y_{0}\right\}\ldots \left\{x_{i},y_{i}\right\}\right\}$, and second, the covariance function, k(x,x') that relates how “close” two points in the domain are to each other—more technically, the degree to which the posterior predictions at two domain points are correlated.

When the covariance function is designed as a nonlinear distance map, the GP covariance function is referred to as a covariance *kernel*, and Gaussian Process inference sometimes framed as a method for estimating the value of unobserved “test” points based on observed “training” points and a kernel that computes distances between training and test points. This view, perhaps more familiar to practicing data scientists, casts Gaussian Process inference in the framework of *supervised learning*. Gaussian Processes are widely used across a variety of real-world domains in part, for their ability to perform well in data-sparse applications (Wang et al., 2005) and for the ready interpretation of their posterior as function estimates with uncertainty bounds.

f(x)∼GP(μ(x),k(x,x')) (1)

### 3.5 Gaussian Processes in Word Space: Empirical Implementation

Gaussian Process can flexibly represent a wide variety of domains and can be tailored by model designers to incorporate domain knowledge *via* the covariance kernel. In this section we will discuss our implementation of this approach, applied to modeling young student’s literacy skills in separate game tasks, defined over a shared domain of English language *words*, called the Curriculum. Because each game task may only be able to access a small amount of personalized data, we leverage the shared domain to perform instance-weighted data *transfer* across game tasks, allowing a model targeting one particular literacy skill (e.g., rhyming) to incorporate personalized data obtained from an interaction focusing on a different literacy skill (e.g., spelling). We also extend this approach to nonstationary environments, by augmenting the conventional Gaussian Process with a “continuous active training data management” protocol, that acts as a mirror to the active learning protocol pursued by the ContentModel–instead of selecting domain points to *add* to the training set based on the GP posterior, the **CATDaM** protocol selects points already *in* the GP training set to *remove* (see Section 3.7)

Over the course of a long-term study (see Section 5 for evaluation details), we learn Gaussian Process models for each game (RhymeRacer and WordBuilder), which we refer to as the StudentModel for that game. Each StudentModel is an estimate of a student’s personal mastery of that game’s intended literacy skill (rhyming or spelling). In our implementation, the StudentModels of both games take the form of a Gaussian Process, defined over the same domain space: a discrete set of *words* (called the Curriculum), a set of 74 words selected in partnership with experts in childhood literacy, supplemented with age-appropriate words to complete the rhyme groups.

Under the framework of supervised learning, the GP mean function is conventionally set to 0 everywhere, leaving the covariance as the primary way for researchers to encode domain knowledge in the model “design”. In fact, because the two task StudentModels differ only in their covariance kernels (they share all other hyperparameters), the covariance functions functionally *distinguish*, and therefore *define* the game task (with respect to each other). In other words, because the two StudentModels share an input space, mean function, and noise hyperparameters, the difference in their posterior estimates, if provided with the same training data, is *solely driven* by the differences in their covariance functions.

The training data take the form of a “target word” from the curriculum and a score, representing an estimate of skill mastery applied to the target word, derived from gameplay. Scores range from (−1, 1), where −1 represents complete lack of mastery, 1 represents full mastery, and 0 represents neutral mastery, providing an intuitive scale for interpreting training data and, hence, the GP posterior. Gaussian Processes can handle a continuous range of inputs, but RhymeRacer and WordBuilder game inputs give only a binary “correct” or “incorrect” answer signals. To map from the binary signal of response correctness, we blend that information with continuous contextual features like timing. The final score ($y_{i}$) for a round Target word (i.e., a “sample”) ($x_{i}$), is derived by combining adding a timing adjustment, $p\left(t_{d}\right)$, to the “correctness” binary variable (1 or 0), to correct for the possibility of guessing. The timing adjustment is applied as a discrete, step-wise penalty of 0.1 based on the number of seconds it takes to give an answer i.e, $p\left(t_{d}\right)=0.1\cdot t_{d}$ where $t_{d}$ is the time of delay in seconds. For example, if a student selects the correct Prompt word for a round within the first second, they receive no penalty, but if they selected the correct Prompt word after 5 s, they receive a penalty of $p\left(t_{d}\right)=0.5$. The timing values are scaled differently in WordBuilder, but follow the same procedure and, ultimately, are converted to the same range before instance-weighted transfer.

#### 3.5.1 Designing RhymeRacer and WordBuilder Covariance Functions: A Gaussian Process Example in Word-Space

The key difference between the two game StudentModels is their *covariance* kernels, which compute a distance metric between words in the Curriculum, bringing pairs of words “closer” together when their task outputs (i.e., estimated student mastery) are more highly correlated. In RhymeRacer, the covariance function is based on the cosine distance between the GloVe semantic word vectors (Pennington et al., 2014) of each domain word, plus an additional term that increases the covariance between two words which share a final rhyme ending (i.e., when words are part of the same rhyme group) (Eq. 2). This combination of semantic and phonetic information has previously been validated by in-person student studies (Spaulding et al., 2018), and was developed with input from external collaborators with expertise in early language and literacy skill development.

$$Cov_{rr}\left(\left\{w_{i},w_{j}\right\}\right)=\nu \left[\alpha +\cos\left(GloVe\left(w_{i}\right),GloVe\left(w_{j}\right)\right)\right],$$ (2)

where *α* = 1.0 iff $w_{i}$ and $w_{j}$ share a rhyme ending, and 0 otherwise. *ν* is a normalization constant.

WordBuilder’s covariance function, reflecting the spelling-oriented is based on *orthographic* information—information about the letters that make up a word’s written form. The foundation of the covariance kernel is the Levenshtein distance, normalized over the combined length of the two words (Eq. 3). In essence, this kernel reflects the idea that words which require fewer letter additions, deletions, or substitutions to convert one word to the other are more likely to be mastered together, or not. In both tasks, the covariance kernels primarily function to help the GP StudentModels quickly generalize from observed samples to words not yet seen in the curriculum, improving the efficiency of model learning, as well as enabling the ContentModel to make personalized choices about which words from the curriculum to introduce in the games.

$$Cov_{wb}\left(\left\{w_{i},w_{j}\right\}\right)=\nu \left[Levenshtein\left(w_{i},w_{j}\right)\right],$$ (3)

### 3.6 Transferrable Gaussian Processes: An Instance-Weighting Protocol Based on Task Covariance Similarity

Both WordBuilder and RhymeRacer models work well on their own as single-task models (see Section 5.1 for single-task baselines), but the broader goal of this project is to *transfer* observed training data from one game’s StudentModel to a StudentModel targeting the other game, i.e., multitask personalization.

Both games’ models share the same underlying Gaussian Process form, defined over a word space from the Curriculum. Unique to each game task is the *geometry* of this space, defined by the respective covariance kernels. How should we leverage this unified representation to transfer data from a source task to a target task (and back)? Because the two tasks are broadly related (i.e., both early literacy skills, and individual mastery likely correlated between them), we could consider simply adding all observed source task data to the target task training set. However, this approach ignores that some source task data points are more informative to the target task than others. In other words, the correlation of source task output with target task output *varies over the word space domain*. Moreover, we can use the definitions of the covariance kernels to compute a metric of task similarity at each domain input point, which gives us a score of how similar the local geometries are for each task. We can interpret this instance-specific task similarity metric as a *transfer coefficient*. “Instance-weighting” refers to a family of transfer learning methods that training a target task model on source task data, where the source-task data is re-weighted (a very simple form of task-transformation) before incorporation into the target-task training set (Pan and Yang, 2009). Thus we describe our transfer learning approach as an instance-weighting method, where each instance’s transfer coefficient is derived from a similarity metric between each word’s use in one game and its use in another (Eq. 4).

In prior work, we described the intuition behind this approach as such:

“The covariance function of RhymeRacer encodes the domain knowledge that words which share a rhyme ending are “closer” to each other (i.e., if you can correctly identify the rhyming word for DOG, you are more likely to be able to identify the rhyming word for FROG) (Lenel and Cantor, 1981). Likewise, the covariance function of WordBuilder encodes the domain knowledge that words which share similar letters are “closer” to each other (i.e., if you can correctly spell CAT, you are more likely to be able to spell CAR). When computing the instance weight of ‘(DOG, 0.85)’, if knowing DOG impacts the inference of other words in the source task in a way similar to how knowing DOG impacts inference in the target task, then DOG should be weighted roughly equally (i.e. close to 1) in the target task. More concisely, the greater the source-target similarity in word-space geometry around a domain point, the higher the transfer weighting of any source task data at that domain point.

To formalize this intuition, we take the average (over all words in the curriculum) difference between source and target task covariances of the instance word and each other word, giving a measure of how similarly instance word data impacts inference overall in the source and target tasks. Transfer weight, $\lambda_{i}$, of a source task data instance $\left\{x_{i},y_{i}\right\}$ is determined by the average difference in source and target task covariance at that point, across all words *w* in the Curriculum, *W*” (Spaulding et al., 2021)

$$\lambda_{i}=\frac{\sum_{w\in W}1-||Cov_{s}\left(x_{i},w\right)-Cov_{t}\left(x_{i},w\right)||}{\left|W\right|}$$ (4)

A transfer coefficient of 1 indicates “perfect” transfer i.e., that instance word conveys the same information in both source and target tasks, whereas a transfer coefficient of 0 indicates that the source and target task are uninformative to each other, with respect to that instance word. To avoid undue complications in evaluating this method, we reweight data instances only once in our evaluations, from the originating source task to the target task. If the model switches tasks multiple times, previously transferred data is not re-weighted and re-transferred back to the original source-task model.

By design, the range of possible training data scores lies within (−1, 1), which, in addition to providing a natural interpretation of scores as “mastery”, also simplifies the instance-weighting transfer procedure. Because positive values are interpreted as positive mastery and negative values as lack of mastery, multiplying by the (positive) transfer coefficient *λ* can never change the sign of a training instance i.e., a negative demonstration in RhymeRacer remains a negative demonstration in WordBuilder.

### 3.7 Improving Nonstationary GP Modeling *Via* Continual Active Training Data Management

In this paper, we propose to move from multitask personalization to “lifelong personalization” by extending transferrable personalized models to *nonstationary* domains. We accomplish this primarily *via* a novel extension to the Gaussian Process modeling framework described thus far. In prior work (Spaulding et al., 2018; Spaulding et al., 2021) we noted that GPs do not naturally have a sense of temporal data-if the model receives training points of (0,1) and (0,−1), the mean posterior prediction is indeed (0,0). But, counter to the intuitive interpretation of variance as uncertainty, the posterior distribution at 0 is not a *high variance* Gaussian (indicating a newly uncertain prediction), but rather a low-variance Gaussian (indicating certainty that the “true” value lies in between the observed data points). Unlike other uncertainty-based estimation methods (e.g., Kalman filters) in which uncertainty is updated over time, Gaussian Processes lack a mechanism for increasing uncertainty around previously observed training data. Our solution, aimed at adapting GPs to lifelong learning scenarios, is an active “learning” protocol we call “continual active training data management”, or **CATDaM**.

In its simplest formulation, **CATDaM** is comprised of a data structure that organizes the observed training data temporally, and an active learning algorithm that marks “stale” data points and removes them from the active training set. Much as the active learning method used by the ContentModel (described in Section 3.3) is closely tied to the tutoring agent’s choice to observe student response, the active removal of training data followed by **CATDaM** is closely tied to the tutoring agent’s *demonstrations*.

Demonstrations by the tutoring agent represent the most direct opportunity for the agent to influence *student* learning, by providing the correct response to a prompt word (as a player) and explaining out loud its reasoning to the student. As described in Section 3.3, the agent’s decision to give a demonstration and the Curriculum word demonstrated are, in fact, coordinated by the StrategyModel, the ContentModel, and the StudentModel. The decision to take the DEMONSTRATE strategy action comes first, and then the ContentModel selects the word which the StudentModel is most confident the student has *not* mastered (i.e., has a negative posterior prediction for mastery).

A demonstration represents important contextual information for **CATDaM**! It signals that a student’s mastery with respect to that domain point (i.e., their mastery of the demonstrated word) may have shifted, and that prior observations of student performance may no longer reflect their current mastery. In order to address this potential distribution shift in student mastery, **CATDaM** marks prior observations of student response to that target word in the memory data structure and removes them from the active training set. Not only does the **CATDaM** protocol remove training data that may no longer reflect the current “distribution”, but it also has the additional advantage of directly increasing model uncertainty at the demonstrated domain point, signaling to the ContentModel that it is a good candidate for observing student performance at a future opportunity.

In the following sections, we evaluate the effect of adding **CATDaM** to a StudentModel through a series of simulation experiments.

## 4 Simulated Students: Pre-Study Evaluation for Long-Term HRI Systems

Although uncommon, it is by no means a new idea within HRI to simulate human data to evaluate robot behavior, models or algorithms under gentler (and more repeatable) conditions. The benefits of this practice are most clearly articulated in a paper that describes the “Oz of Wizard” paradigm, inverting the better-known “Wizard of Oz” paradigm in which real humans interact with a robot whose behavior is actually produced by a human (Steinfeld et al., 2009). Under the Oz of Wizard paradigm, real robot behavior is evaluated against humans whose behavior is actually produced by a computer, i.e., *simulations* of human behavior. “Oz of Wizard” experiments involving “simulated” students are rarely publicized, despite the widespread use of simulators in other areas of robotics (e.g., Sim2Real motion planning or task learning). In part this is because real student behavior is not easy to simulate. Real students act unpredictably, capriciously, and in ways that even the students themselves struggle to articulate.

In many fields of engineering where the “actual” live test of a system is expensive, overly time-consuming, or carries substantial risk, simulation studies are considered *de rigeur*. Despite a simulation fidelity gap larger than many physical environment simulations, we believe simulated student evaluations can advance research in long-term human-robot interactions by providing a more principled starting point for systems prior to conducting long-term in-person studies. For instance, studies on simulated student data can confirm that modeling algorithms perform as expected on simplified data distributions, simulated student data can also help algorithm designers tune hyperparameters to useful values or establish reasonable performance baselines without having to conduct pilot tests on live students. They can also allow for many different comparisons to be made in parallel, whereas human-subjects studies are more typically tightly controlled owing to the generally small number of participants, which has the unfortunate side effect of limiting the number of hypotheses that can be evaluated. We believe that the use of simulated student tests should not be considered a *substitute* for an in-person evaluation, but rather an important and insightful part of the system implementation and preparation before a study of in-person long-term interaction is launched.

In a 2021 review published in the Proceedings of the National Academy of Sciences, roboticists highlighted HRI as an area where simulation has great potential, but also faces many challenges.

Development of simulation tools that better represent the psycho-social nature of HRI and enable a common operating “picture” of possible solution sets for decision making may … establish a baseline for more effective collaboration … Creating (simulated human) avatars is as difficult as humans are diverse, each person a unique and complex web of intertwined physical, social, emotional, cognitive, and psychological threads … Numerous questions remain unanswered in relation to abstracting in mathematical models the psychological underpinnings that trigger in humans states of anxiety, fear, comfort, stress, etc. In this context, the ability to control and display emotions in (simulated human) avatars represents a prerequisite for endowing smart robots with a sense of empathy in their interaction with humans (Choi et al., 2021)

### 4.1 Simulating Student Performance Data

In this section we describe how we derive simulated student performance data, which we use to analyze the effects of multitask personalization through cross-task model transfer and a “lifelong personalization” extensions *via* **CATDaM**. We outline our implementations of two classes of simulated “students” (referred to as “simple” and “dynamic” SimStudents), describe the theoretical assumptions on which these simulations are based, and discuss the implications of subsequent simulation experiments. Sections 4.1.1–4.1.3 are reprinted from (Spaulding et al., 2021). The implementation of the simple SimStudent is unchanged from that prior work in the new experiments and results reported here.

#### 4.1.1 SimStudent: A Sketch of Student Behavior for HRI Simulations

Each SimStudent has an internal “true mastery” ($m_{w}\in \left[-1,1\right]$) for each word in the Curriculum, per game. The SimStudent’s true mastery of a word in a game can be interpreted as the student’s likelihood of correctly applying the literacy skill to the word (e.g., identify “SNAIL” as the rhyme for “WHALE” or correctly spell “SNAIL” with the letter blocks). The process for generating true mastery values varies by game, and is used to simulate a student’s gameplay actions during the game *via* a noisy sampling process.

Each SimStudent’s “performance data” for a word consists of a binary “correctness” variable corresponding to whether they successfully applied the primary literacy skill of the game to the word (e.g., selected the correct rhyme or correctly spelled the Target word), plus a scalar “timing” variable corresponding to the amount of (simulated) time taken to answer. Each word-performance pair ($word_{i},\left\{correct_{i},timing_{i}\right\}$) constitutes a single “sample”.

#### 4.1.2 Simulating True Mastery

Although each game supports the practice of different fundamental literacy skills (rhyming and spelling), both skills are indicators of a meta-linguistic skillset known as *phonological awareness*. To generate the SimStudent’s true mastery of each word in each game, we first generate a theoretical “phonological” mastery for each of the 39 ARPAbet phonemes (Hixon et al., 2011), uniformly at random ($m_{p}\in \left[-1,1\right]$). The phonological mastery that underlies the word-mastery of both games is an implicit modeling assumption, based on decades of research in early childhood literacy development, that there exists a link between a student’s rhyming and spelling ability with respect to specific words and phonemes (Høien et al., 1995). After random initialization, these phonological mastery values are then further transformed to derive the mastery of each Curriculum word in each game. For RhymeRacer, the mastery of the phonemes that comprise each rhyme-ending (e.g., “AY”-“N” for “RAIN”, “BRAIN”, and “TRAIN”) are averaged, and Gaussian noise (centered on the phoneme-mastery mean, σ=0.1) is independently added to compute the SimStudent’s true mastery of each word with that rhyme-ending. For WordBuilder, the phonological mastery of all phonemes that constitute a word are averaged to give the SimStudent’s true mastery of that word.

#### 4.1.3 Simulating Performance Data From Mastery

The “correctness” component of student performance is determined by whether the student’s true mastery of that word is greater or less than 0 (corresponding to correct/incorrect). However, the value of this component is randomly flipped at a rate equal to “guess” and “slip” binomial variables. “Guess” and “slip” parameters are common formulations in educational student modeling research (Baker et al., 2008), which we use here to make our simulated student data more realistic. Respectively, guess and slip parameters correspond to the probability of *correctly* answering a question *without* true mastery or *incorrectly* answering a question *despite* true mastery. For RhymeRacer, we set guess and slip rates at 0.25 and 0.1, based on the multiple-choice nature of the round gameplay. For WordBuilder, due to a game design less conducive to successful guessing, the guess and slip rates are set at 0.1 and 0.1.

The “timing” component of student performance is determined by the numerical value of the SimStudent’s true mastery, mixed with Gaussian noise. For these experiments, we capped the maximum timing at 10 s. The student’s true mastery score is binned into deciles, and the final score is calculated by sampling from a Gaussian centered on 10−MasteryDecile, so that lower levels of mastery correspond to longer timing components.

### 4.2 Dynamic Students

The dynamic SimStudent largely keeps the same implementation as the simple SimStudent, and extends it by adding a *learning rate* and a *learning gain* parameter. Whenever the tutoring agent gives a demonstration, the learning rate parameter determines the probability that the student’s mastery increases, simulating student learning. The magnitude of the score rise in the student’s underlying word mastery is set by the learning gain parameter (word mastery is capped at 1, and further student learning from tutor demonstrations has no effect). In the experiments reported here, the learning rate was set to 0.66 and the learning gain was set to 0.50 (so if mastery were at its lowest possible value, two successful lessons would be sufficient to boost mastery to halfway, and four successful lessons would boost mastery to its highest value). Other than the probabilistic shift in word mastery in response to tutor demonstrations, the dynamic SimStudent’s word mastery and performance data are simulated identically to the simple SimStudent.

## 5 Evaluating Lifelong Gaussian Processes and Multitask Transfer in Simulation

Our primary research goal with this paper is to extend prior results on multi-task personalization to a more realistic simulation scenario that emphasizes the non-stationary nature of a student learner during a tutoring interaction. Therefore, we will quickly recap prior results on multitask personalization in the stationary case (simple SimStudent), then explore how introducing robot demonstrations, student learning updates, and the addition of continuous active training data management (**CATDaM**) affects our evaluation of multi-task personalization.

The primary questions we were interested in answering with this work were fundamental measures of transfer learning systems: *viability*, *proficiency*, and *efficiency*. In other words, 1) Viability: does incorporating source task data improve target task performance at all, or do we find that source task data is worse than no data i.e., negative transfer? 2) Proficiency: Does a target task model trained on source and target task data perform better than a target task model trained on the same amount of *total* data, exclusively from the target task? 3) Efficiency: Does a target task model trained on source and target task data perform better than a target task model trained on the same amount of *target task* data only?

Our primary metric for evaluating models is the F-1 classification score, which combines precision and recall. The classification task is whether the model correctly predicts the *sign* (i.e., positive or negative) of the SimStudent’s true word mastery. While this may seem a coarse metric for simulated study—we could, for instance, look at L1 or L2 regression loss—the sign of the word mastery is the primary determinant of the correctness of the student’s response (guesses and slips notwithstanding). In a study with real students, we do not have access to a numerical form of a student’s “true” mastery; student models are evaluated based on their ability to predict student’s actual response behaviors. Therefore, in the spirit of keeping our simulation as close as possible to human subject study, we focus our evaluation on the same metric: binary classification of student mastery with respect to individual curricular components.

Each figure below shows the results of the average of 20 “rollouts” of 60 “samples” for each of three classes of model: RhymeRacer single task, WordBuilder single task, and a transfer model (color shading indicates standard error of the mean). At the start of each rollout, a new SimStudent (with newly randomized word mastery) is created to represent a unique student. Each rollout consisted of 60 samples, intended to mirror the structure of many common studies of long-term interactions—four interaction “sessions” each of which provided 15 useful samples [roughly in line with the actual number of samples collected in live human-robot experiments reported in Spaulding and Breazeal (2019). Within each rollout, the transfer model alternates tasks at the start of each “session” i.e., after 15, 30, and 45 samples respectively. Within each rollout, “samples” represent opportunities for the tutoring agent to OBSERVE students mastery *via* game performance.

In live gameplay, the StrategyModel determines whether the tutoring agent DEMONSTRATEs or OBSERVEs. For our simulation study, we adopt a simple rule-based StrategyModel: the robot chooses to DEMONSTRATE after a fixed number of samples and OBSERVE otherwise. In the case of a typical 60 sample rollout, the tutoring agent DEMONSTRATES twice after every three samples it OBSERVEs, starting after the first nine samples. So in a 60 sample rollout, the student receives 34 “demonstrations” from the robot (not all of which result in successful learning), 2 each after 9, 12, 15 … samples.

**FIGURE 5:**
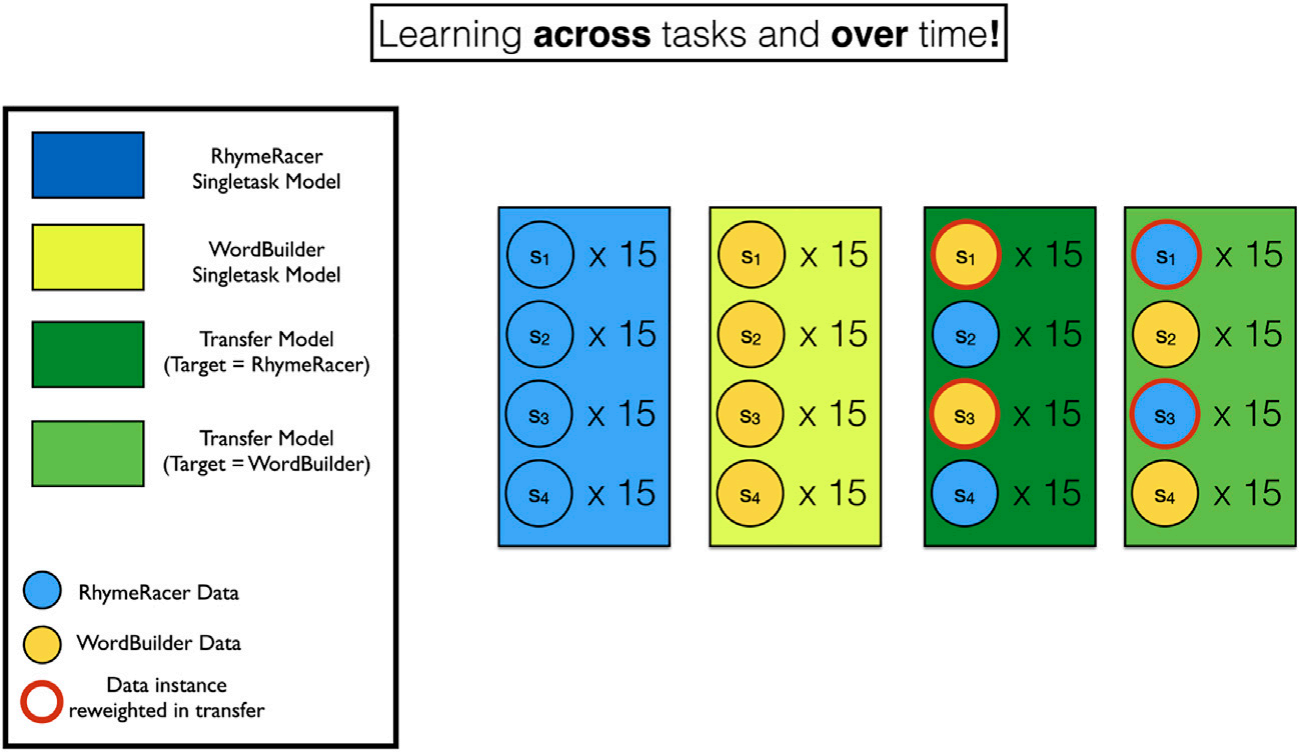
Visual depiction of training data for single- and multi-task student models. Blue and Yellow rectangles and circles indicate models and data instances from RhymeRacer and WordBuilder. Red rings indicate data has been re-weighted from its originating source task to a new target task.

Figure 5 shows the structure of the training data for each class of model graphically.

In live gameplay, the StrategyModel determines whether the tutoring agent DEMONSTRATEs or OBSERVEs. For our simulation study, we adopt a simple rule-based StrategyModel: the robot chooses to DEMONSTRATE after a fixed number of samples and OBSERVE otherwise. In the case of a typical 60 sample rollout, the tutoring agent DEMONSTRATES twice after every three samples it OBSERVEs, starting after the first nine samples. So in a 60 sample rollout, the student receives 34 “demonstrations” from the robot (not all of which result in successful learning), two each after 9, 12, 15 … samples.

### 5.1 Multitask Personalization With Stationary Students

**FIGURE 6:**
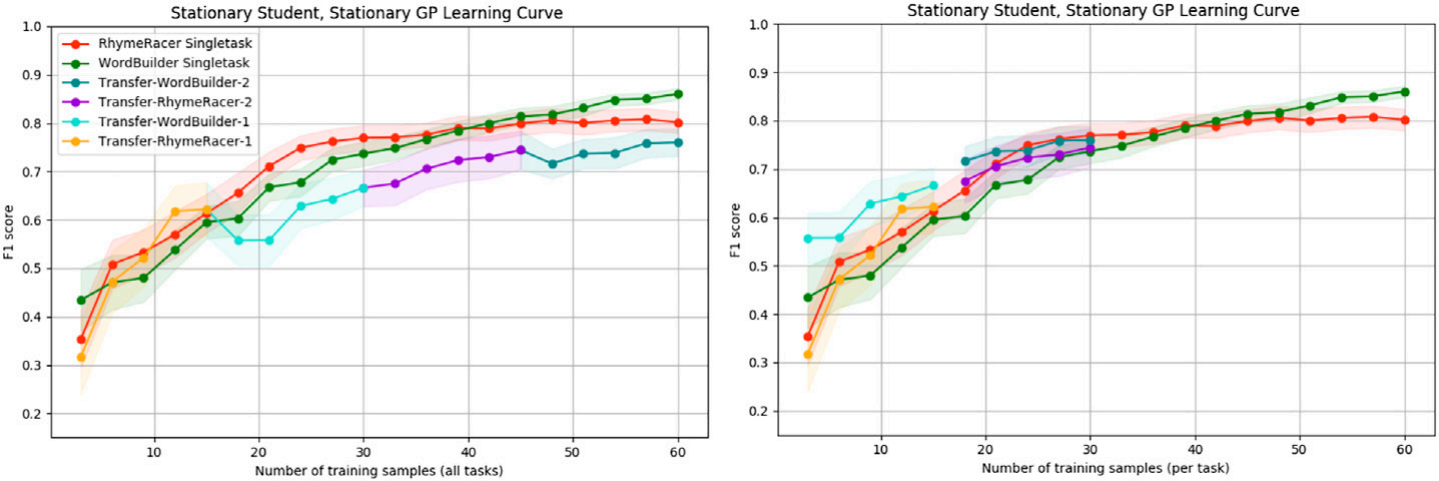
Simple “Proficiency” and “Efficiency” evaluation of multi-task vs. single-task personalized models when RhymeRacer is the first task. The transfer model trades off final classifier accuracy for multi-task generality and meets or exceeds single-task model performance with equal amounts of target task data.

**FIGURE 7:**
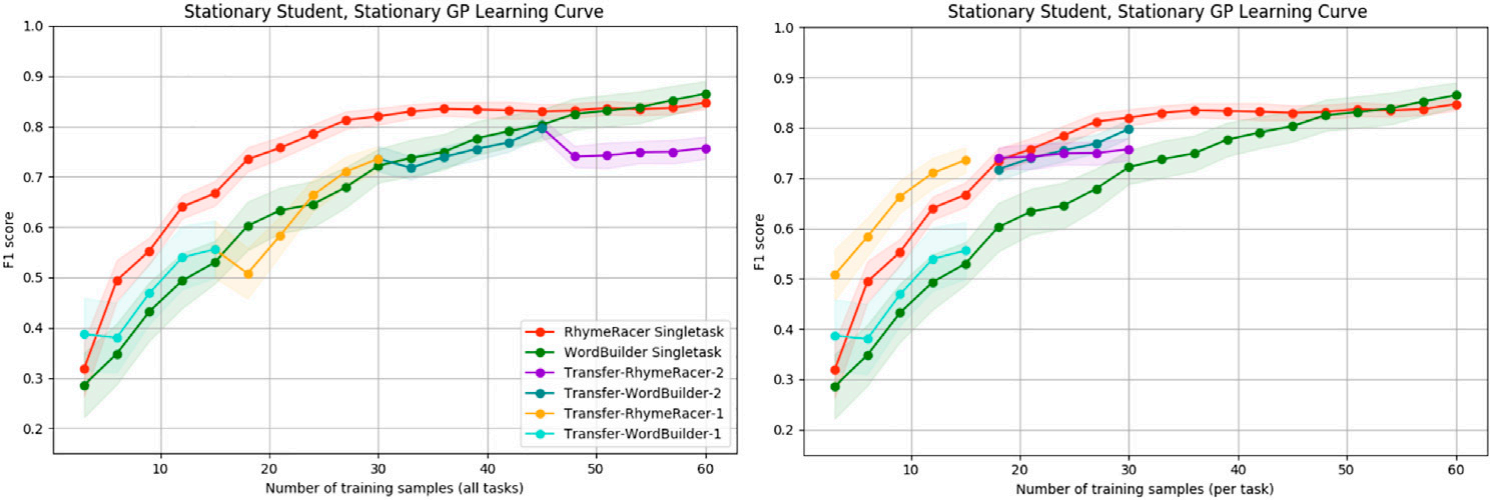
Simple “Proficiency” and “Efficiency” evaluation of multi-task vs. single-task personalized models when WordBuilder is the first task. The general trend is consistent with the results when RhymeRacer is first, indicating that the results are stable independent of task order.

In prior work, we showed that multitask personalization can improve the efficiency of target task model learning, and that this effect is most pronounced within the first few samples collected during an interaction. This is a critical step towards reducing the problem of cold-start learning in interactive machine learning. The results in this section were previously reported in Spaulding et al. (2021). Here, we give further context for these results and provide new supporting evidence to support their conclusions, showing that the effect persists even when the task order is reversed. Figure 6 shows the results of the rollouts when RhymeRacer is the starting task, and Figure 7 show the corresponding results when WordBuilder is the starting task.

First, we discuss the single-task models. Both single-task models learn good representations of their respective game tasks over 60 samples, consistent with prior experimental results (Spaulding and Breazeal, 2019), suggesting that our simulation settings are reasonably implemented, giving confidence in further results not yet evaluated in an experimental setting with human students.

The transfer model data is depicted in two separate representations, each of which is better suited to answering different questions. The “continuous” representation (left, both figures) shows the transfer model data as a single rollout of 60 samples, with each session segment colored to show transfer. This representation is best suited for exploring questions of final proficiency—how well do transfer models trained on a mix of source and target task data compare to single-task models trained on the same amount of data exclusively from the target task? The “discontinuous” representation (right, both figures) shows the transfer model data split into discrete session segments, with their position on the *x*-axis determined by the amount of *target* task data. This representation is best suited for exploring questions of model efficiency—how well do transfer models trained on a mix of source and target task data compare to single-task models trained on the same amount of data exclusively from the target task?

Figure 6 shows that initial transfer from RhymeRacer to WordBuilder is substantial and positive, and that a WordBuilder model trained on prior data from RhymeRacer outperforms a single-task WordBuilder model, particularly during crucial early interaction rounds. Figure 7 shows that the effect remains consistent when the task order is reversed (i.e., when the task sequence starts with WordBuilder). In this case, we can see that transfer from WordBuilder to RhymeRacer boosts initial performance, but that subsequent transfer effects are less impactful as more target task data is gathered, suggesting that the benefits of task transfer may not be perfectly reflexive (i.e., the benefit of transferring RhymeRacer data to WordBuilder may not be equal to the benefit of transferring data from WordBuilder to RhymeRacer).

Overall, these results from the simplified simulation environment paint a compelling enough picture to merit further investigation of multitask personalization in the nonstationary setting. Positive transfer is evident in both directions, and there is strong evidence that multi-task personalization is most impactful in crucial early phases of an interaction, before a model has an opportunity to acquire significant target-task training data.

### 5.2 Lifelong Personalization With Dynamic Students: Effects on Model Proficiency and Data Efficiency

Now we turn our attention to evaluating qualities of multitask personalization in a more complex, nonstationary simulation scenario that incorporates the effects of a tutoring agent’s actions on dynamic (i.e., learning) students. In these evaluations, results for all models were derived over the same simulation timeline of 60 samples, even though in studies with real students, there is often a trade-off between opportunities for the tutoring agent to respond (“demonstrations”) and the student to respond (“samples”). Because we are primarily interested in understanding data and performance trade-offs between different kinds of computational models, we chose to evaluate them over consistent data sample timelines. Even though the models evaluated with a dynamic student incorporate demonstrations and student learning and models evaluated on static students do not, we evaluate them both with respect to the same 60 sample timeline. We also provide new results from adding “continuous active training data management” (**CATDaM**) to the GP StudentModel for “lifelong” learning, and present evidence that including **CATDaM** can improve both model performance and student learning in nonstationary scenarios.

First, we show what happens when we apply the original, static Gaussian Process model (without **CATDaM**) directly to a nonstationary simulation with agent demonstrations and dynamic students.

**FIGURE 8:**
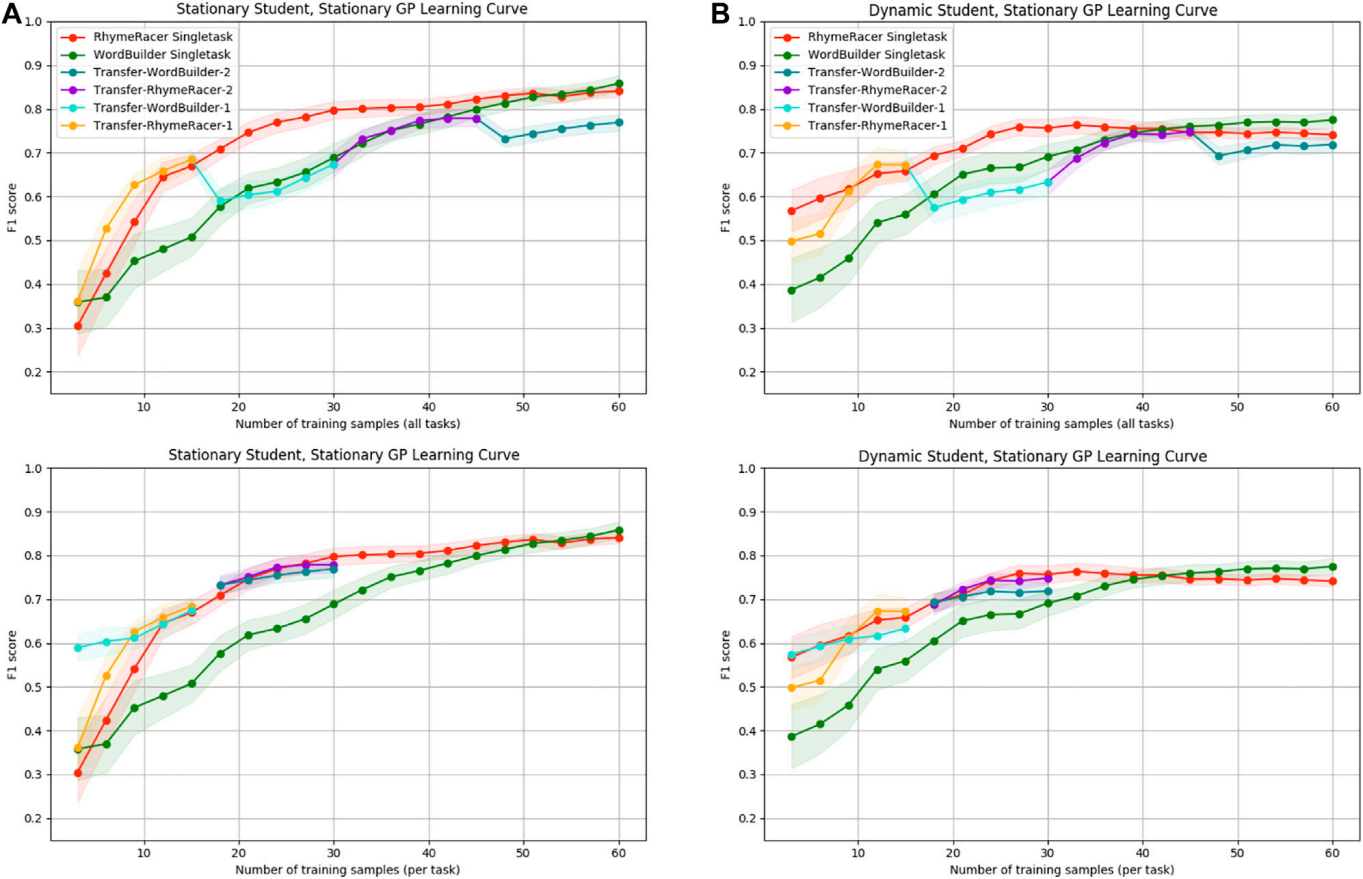
Static-model-static-student performance results **(A)** vs. static-model-dynamic-student performance results **(B)**. Static models can learn a decent model but suffer a drop in final proficiency. Efficiency benefits of multitask model are undiminished.

Figure 8 compares static single-task and transfer models evaluated in two different scenarios. On the left, we have the same experimental conditions as Figures 6, 7, in which underlying student performance is derived from a static SimStudent and there are no demonstrations to promote student learning. On the right, the modeling GPs take the same modeling approach, but the underling student performance data is derived from dynamic SimStudents. Demonstrations from the tutoring agent slowly cause shifts in underlying student mastery. This shows the expected performance gap from modeling a dynamic target using non-stationary modeling approach. Even under these more challenging conditions, the GP modeling framework can still learn a passable student model, but on the right, we see the relative impact of “stale” data and student mastery shift impede performance. Across all classes, final model proficiency stabilizes at an F1-score of (0.74–0.79), compared to (0.84–0.87) when modeling static students, a drop of 10 percentage points. The final proficiency of the multitask model also declines across both tasks, though the performance loss is less than in the single-task case. Despite this hit to overall proficiency, the most notable trends of the multitask transfer model, positive transfer and early-sample efficiency gains, remain.

**FIGURE 9:**
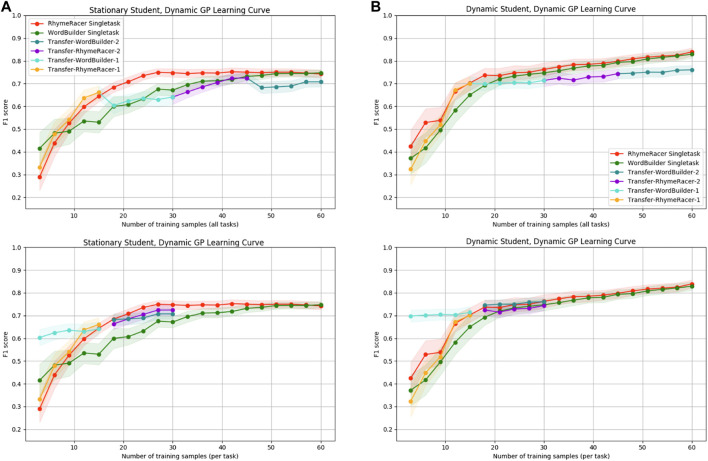
Static-model-dynamic-student performance results **(A)** vs. dynamic-model-dynamic-student performance results **(B)**. Adding **CATDaM** to GP models improves modeling performance in nonstationary environments, while preserving efficiency benefits of multitask personalization.

Next, we examine the benefits of incorporating continual active training data management (**CATDaM**) into Gaussian Process student models.Figure 9 compares static single-task and transfer models (on the left) to lifelong (i.e., uses **CATDaM**) single-task and transfer models on the right. For both classes of model, underlying student performance is derived from a dynamic SimStudent that receives demonstrations.

On the left, we see the same general performance trend as the right side of Figure 8. The static GP model learns a decently performant model of the dynamic student, but student learning causes both single-task and multitask models to quickly hit a lower performance ceiling than in the static-student-static-GP case. Without accounting for shift in dynamic student mastery, the learning curve for static-GP models flattens and even declines slightly. On the right, it continues to rise throughout the full 60 sample rollout, hitting basically the same level of performance as the “static-student-static-GP” case from Figure 8.

To summarize these results: when we increase the complex and realism of the simulation environment by adding in a non-stationary SimStudent and tutor demonstrations, stationary GP models perform about 8–10 percentage points worse (10–15%). Augmenting the GP model with **CATDaM** helps the Gaussian Process better model the non-stationary effects of tutor demonstrations, and performance performs as well in a more complex, nonstationary environment as a stationary model does in a stationary environment.

And, while non-stationarity lowers the final proficiency of static GP models, it does not appear to materially impact the *efficiency* results from multitask transfer. Nor are efficiency results clearly impacted when GP model proficiency rises as a result of incorporating **CATDaM**. This result that the efficiency benefits of a multitask personalization approach are independent of the *proficiency* benefits of a continual learning approach.

### Lifelong GP Modeling of Dynamic Students: Effects on Student Learning

**FIGURE 10:**
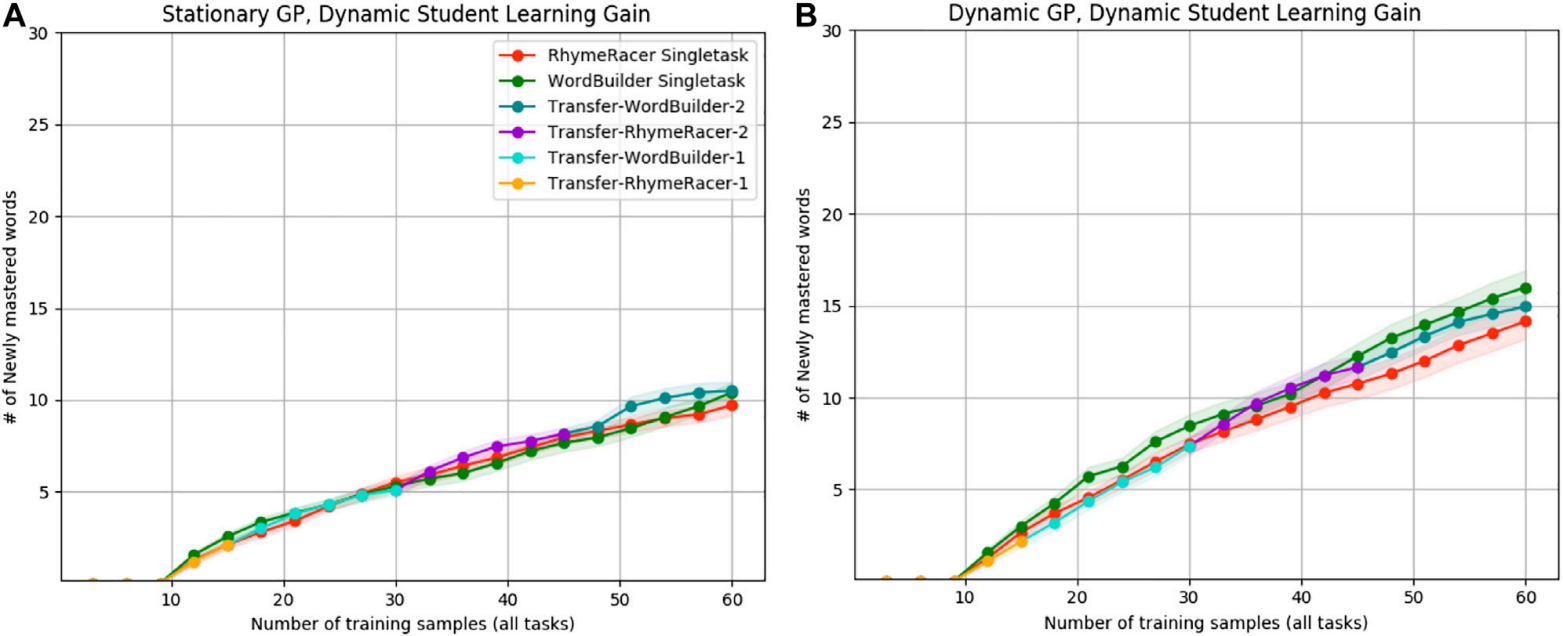
Student learning gains under static-model-dynamic-student **(A)** and dynamic-model-dynamic-student **(B)** simulations. Students tutored by a dynamic GP model mastered nearly 50% more words.

In addition to enabling more sophisticated evaluation of proficiency and efficiency of personalized model learning, by integrating tutoring agent actions and dynamic student learning into our simulation experiments, we can also study the effect of **CATDaM** on *student learning*, the increase in mastery due to the tutoring agent demonstrations. We quantify these results by calculating the number of “newly mastered” words (mastery went from negative to positive) for each model type over rollouts guided by both static and dynamic GPs. Figure 10 shows that SimStudents in the dynamic GP case learned five more words on average, compared to students in the static GP rollouts. We hypothesize this result is due to the dynamic GP picking “better” words to demonstrate, on account of a more up-to-date estimate of word uncertainty enabled by **CATDaM**.

## Conclusion and Further Discussion

Throughout these experiments, we have strived to carefully contextualize the results as supporting evidence in support of future in-person studies with human students. There is truly no substitute for actual human experimental data. At the same time, we think that these results provide confidence to proceed with live student studies, and are demonstrative of the kinds of benefits for long-term HRI that can come from simulation analysis. When previously introducing this evaluation framework, we wrote:

“In advocating for researchers to evaluate their systems in the real world, Rodney Brooks famously quipped “simulations are doomed to succeed” (Brooks and Mataric, 1993). We find this philosophy generally laudable, if not always practical. Simulated human data has an accepted role in Human-Robot and Human-Agent Interaction research (with notable examples in human-interactive machine learning systems) (Griffith et al., 2013). While this project meets the criteria for such a design, we wish to state that this project constitutes *an* evaluation of the proposed transfer method, it is not a *definitive* evaluation. Further research with human subjects will be necessary, not least, because one of the major hypothesized benefits of the multi-task personalization paradigm—increased student engagement—could not be realistically evaluated by simulation experiments.”

In summary, the main contribution of this paper is extending our prior study of multitask personalization to encompass more realistic aspects of human-robot tutoring interactions, chiefly that student learning is a nonstationary target for cognitive modeling when the robot tutor is actively teaching. To investigate this more complex scenario, we introduced a method for our Gaussian Process transfer learning approach to better handle nonstationary targets, adding a continual active training data management (**CATDaM**) mechanism to each task model. This memory-based active data management allows each model to proactively prune away “stale” data from its training set based on interactive context features (in this case, based on which words the agent provided demonstrations for).

Not only does extending our evaluation of multitask learning to more realistic non-stationary domains lend further confidence that simulation results will extend to live long-term studies with students, but the addition of robot actions (demonstrations/observations) and stochastic student learning updates also allows us to analyze estimated student learning gains in simulation. We found that in simulated interaction with a tutoring agent using a **CATDaM**-enabled model leads to a simulated learning gain of almost 50% more new words mastered, compared to an agent using a static StudentModel. These results are consistent over both single-task and multi-task models, and are robust to task order in the multitask case.

We also find evidence that the extension of our modeling approach to nonstationary domains does not alter the positive transfer benefits of data efficiency and cold-start avoidance previously observed in evaluating multitask personalization. In other words, adopting a continual learning approach appears to be *complementary* to a multitask personalization approach. Finally, we show that adopting a continual learning approach to dynamic student modeling also has benefits for *student learning* in addition to model learning.

Overall, these results paint a bright picture for future research in long-term HRI. Combining multitask personalization and continual learning into a “lifelong personalization” approach appears to benefit both the data efficiency of model learning, the final proficiency of learned student models, and the amount of student learning gain. The simulation experiments presented here provide useful insight as technical validation in advance of a long-term in-person study, and may also prove useful in persuading institutions to engage in long-term HRI research as a scientific partner. Naturally, more research will be needed to confirm these effects in studies with real students. But these results bring us one step closer, providing compelling evidence that combining continual learning and multitask personalization can be a successful path toward truly lifelong personalized companions.

## Data Availability

The raw data supporting the conclusions of this article will be made available by the authors, without undue reservation.
